# In the national epidemiological bulletins – a selection from recent issues

**DOI:** 10.2807/1560-7917.ES.2018.23.26.1806281

**Published:** 2018-06-28

**Authors:** Eurosurveillance editorial team

**Affiliations:** 1 European Centre for Disease Prevention and Control (ECDC), Stockholm, Sweden

**Keywords:** infectious diseases, public health, Europe

**Healthcare-associated infections and antibiotic resistance**

 

Did we get his message right? A short history of hand hygiene on the occasion of the 200th anniversary of Ignaz Semmelweis’ birth

Epidemiologisches Bulletin 18/2018

3 May 2018, Germany

https://www.rki.de/DE/Content/Infekt/EpidBull/Archiv/2018/Ausgaben/18_18.pdf?__blob=publicationFile

 

*Clostridium difficile* outbreak investigations

Epidemiologisches Bulletin 14/2018

5 April 2018, Germany

https://www.rki.de/DE/Content/Infekt/EpidBull/Archiv/2018/Ausgaben/14_18.pdf?__blob=publicationFile

 

**Food- and waterborne diseases**

 

RKI guidance on *Campylobacter* infections

Epidemiologisches Bulletin 23/2018

7 June 2018, Germany

https://www.rki.de/DE/Content/Infekt/EpidBull/Archiv/2018/Ausgaben/23_18.pdf?__blob=publicationFile

 

Shigellosis outbreak at a student accommodation site

Infectieziekten Bulletin 2018; 29(6)

June 2018, the Netherlands

https://magazines.rivm.nl/2018/06/infectieziekten-bulletin/uitbraak-van-shigellose-bij-een-studentenvereniging

**

*Salmonella* and *Campylobacter* infections 2016-17

EPI-NEWS 15/2018

11 April 2018, Denmark

https://www.ssi.dk/English/News/EPI-NEWS/2018/No%2015%20-%202018.aspx

 

Giardiasis in a nursery

Vlaams Infectieziektebulletin, 4/2017

2017, Belgium

https://www.zorg-en-gezondheid.be/sites/default/files/atoms/files/VIB%202017-4_DEF.pdf

 

**Hepatitides**

 

Special edition: National day against viral hepatitis

Bulletin épidémiologique hebdomadaire, 11/2018

15 May 2018, France

http://invs.santepubliquefrance.fr/beh/2018/11/pdf/2018_11.pdf

 

Hepatitis A outbreak in a care home

Infectieziekten Bulletin 2018; 29(4)

April 2018, the Netherlands

https://magazines.rivm.nl/2018/04/infectieziekten-bulletin/hepatitis-uitbraak-op-een-zorgboerderij

 

**Vaccine-preventable diseases**

 

Laboratory confirmed cases of measles, mumps and rubella, England: January to March 2018

Health Protection Report; 12(19)

1 June 2018, United Kingdom

https://assets.publishing.service.gov.uk/government/uploads/system/uploads/attachment_data/file/711941/1918_AA_mmr.pdf

 

Vaccine coverage for the GP based catch-up meningococcal ACWY (MenACWY) immunisation programme in England to the end of March 2018

Health Protection Report; 12(18)

25 May 2018, United Kingdom

https://assets.publishing.service.gov.uk/government/uploads/system/uploads/attachment_data/file/711444/hpr1818_menACWY-vc.pdf

 

Preliminary vaccine coverage estimates for the meningococcal B (MenB) immunisation programme for England, update from January to March 2018

Health Protection Report; 12(15)

27 April 2018, United Kingdom

https://assets.publishing.service.gov.uk/government/uploads/system/uploads/attachment_data/file/703471/hpr1518_menbVC.pdf

 

Vaccination rates at school entry examination in Germany, 2016

Epidemiologisches Bulletin 19/2018

19 April 2018, Germany

https://www.rki.de/DE/Content/Infekt/EpidBull/Archiv/2018/Ausgaben/16_18.pdf?__blob=publicationFile

 

Laboratory-confirmed whooping cough 2017

EPI-NEWS 16/2018

18 April 2018, Denmark

https://www.ssi.dk/English/News/EPI-NEWS/2018/No%2016%20-%202018.aspx

 

Free measles vaccination for non-immune adults 

EPI-NEWS 13-14/2018

4 April 2018, Denmark

https://www.ssi.dk/English/News/EPI-NEWS/2018/No%2014%20-%202018.aspx

 

Measles in Ireland – weeks 1-12, 2018

Epi-Insight 2018;19(4)

April 2018, Ireland

http://ndsc.newsweaver.ie/epiinsight/1qv34ih6ub810gkzp9yxn5?a=1&p=53187118&t=17517774

 

**Respiratory diseases**

**

The 2017/2018 influenza season

EPI-NEWS 23-24/2018

13 June 2018, Denmark

https://www.ssi.dk/English/News/EPI-NEWS/2018/No%2023-24%20-%202018.aspx

 

Spotlight on Human Parvovirus B19

Epi-Insight 2018;19(6)

June 2018, Ireland

http://ndsc.newsweaver.ie/epiinsight/tflodsptta910gkzp9yxn5?a=2&p=53465132&t=17517804

 

Fatal case in a child related to an influenza outbreak in a kindergarten

Epidemiologisches Bulletin 22/2018

31 May 2018, Germany

https://www.rki.de/DE/Content/Infekt/EpidBull/Archiv/2018/Ausgaben/22_18.pdf?__blob=publicationFile

 

**Sexually transmitted diseases**

 

Syphilis 2017

EPI-NEWS 22, 2018

30 May 2018, Denmark

https://www.ssi.dk/English/News/EPI-NEWS/2018/No%2022%20-%202018.aspx

 

**Zoonoses and vector-borne diseases**

 

Special edition: Lyme borreliosis and other tick-borne diseases

Bulletin épidémiologique hebdomadaire, 19-20/2018

19 June 2018, France

http://invs.santepubliquefrance.fr/beh/2018/19-20/pdf/2018_19-20.pdf

 

TBE: risk areas in Germany (April 2018)

Epidemiologisches Bulletin 17/2018

26 April 2018, Germany

https://www.rki.de/DE/Content/Infekt/EpidBull/Archiv/2018/Ausgaben/17_18.pdf?__blob=publicationFile

 

Hantavirus infections in Germany: a review of the 2017 epidemic year

Epidemiologisches Bulletin 15/2018

12 April 2018, Germany

https://www.rki.de/DE/Content/Infekt/EpidBull/Archiv/2018/Ausgaben/15_18.pdf?__blob=publicationFile

 

Human case of leptospirosis transmitted from a dog

Infectieziekten Bulletin 2018; 29(4)

April 2018, the Netherlands

https://magazines.rivm.nl/2018/04/infectieziekten-bulletin/humane-leptospirose-een-puppy

 

Leptospirosis among participants in an obstacle run, Nijlen 2015

Vlaams Infectieziektebulletin, 4/2017

2017, Belgium

https://www.zorg-en-gezondheid.be/sites/default/files/atoms/files/VIB%202017-4_DEF.pdf

 

Historical aspects of leptospirosis

Vlaams Infectieziektebulletin, 4/2017

2017, Belgium

https://www.zorg-en-gezondheid.be/sites/default/files/atoms/files/VIB%202017-4_DEF.pdf

